# SII as a predictor of mortality in patients with non-ST-segment elevation myocardial infarction and diabetes mellitus

**DOI:** 10.1016/j.plabm.2025.e00476

**Published:** 2025-05-16

**Authors:** Cuiyuan Huang, Jiajuan Yang, Wenqiang Li, Li Liu, Wei Wang, Haiyan Hu, Jing Zhang, Jian Yang

**Affiliations:** a Central Laboratory, The First College of Clinical Medical Science, China Three Gorges University & Yichang Central People's Hospital, Yichang, China; b Hubei Key Laboratory of Ischemic Cardiovascular Disease, Yichang, China; c Hubei Provincial Clinical Research Center for Ischemic Cardiovascular Disease, Yichang, China; d Department of Cardiology, The First College of Clinical Medical Science, China Three Gorges University & Yichang Central People's Hospital, Yichang, China; e Yichang City Centre for Disease Control and Prevention, Yichang, China

**Keywords:** Systemic immune inflammation index, Non-ST segment elevation myocardial infarction, Type 2 diabetes mellitus, Inflammation, Mortality

## Abstract

**Background:**

Systemic immune inflammation index (SII) is an innovative marker reflecting immune and inflammatory responses.

**Objectives:**

To explore the predictive value of SII on the risk of death in patients with NSTEMI combined with T2DM.

**Methods:**

An analysis of 448 patients with NSTEMI and T2DM admitted to our institution between December 2017 and May 2022 was conducted in this retrospective study. SII values were used to divide patients into high and low SII groups and investigate their impact on mortality.

**Results:**

According to the analysis results, elevated SII levels are significantly linked to a poor prognosis in patients with NSTEMI and T2DM. Over an average follow-up period of 22.75 months, 106 (23.7 %) all-cause deaths were recorded. The optimal threshold for predicting death was found to be an SII value of 1384.596 × 10^9^/L through ROC curve analysis. Kaplan-Meier analysis indicated that the survival rates were higher in the low SII group compared to the high SII group (*P* < 0.001). Elevated SII levels were independently linked to increased mortality in patients with NSTEMI and T2DM, according to univariate (HR:3.19, 95 % Cl: 2.18–4.68) and multivariate COX (HR: 2.72, 95 % Cl: 1.81–4.09) regression analyses.

**Conclusion:**

High SII values were strongly associated with mortality in patients with NSTEMI and T2DM. SII serves as a valuable prognostic tool, enhancing the management and prognosis of patients with concurrent NSTEMI and T2DM.

## Highlights

• SII predicts mortality risk in NSTEMI patients with T2DM.
• Elevated SII levels linked to poor prognosis in NSTEMI and T2DM.
• Optimal SII threshold for mortality prediction identified as 1384.596 × 10^9^/L.
• Kaplan-Meier analysis shows higher survival rates in low SII group.
• SII serves as an independent predictor of mortality in NSTEMI and T2DM patients.

## 1 Introduction

Cardiovascular disease has emerged as the leading cause of mortality globally, imposing a substantial burden on both patients and their families [1]. Acute coronary syndromes (ACS) are characterized by a sudden reduction in blood supply to the heart and include ST-segment elevation myocardial infarction (STEMI), non-STEMI (NSTEMI) and unstable angina. NSTEMI constitutes 70 % of acute coronary syndromes attributable to coronary artery disease [2]. Diabetes mellitus, a significant risk factor for coronary heart disease, has seen nearly a twofold increase in prevalence over the past three decades, with the majority of patients diagnosed with type 2 diabetes (T2DM). Studies have identified that up to 32 % of individuals with T2DM develop coronary heart disease [3]. Moreover, research has demonstrated that T2DM significantly influences the long-term prognosis of patients diagnosed with NSTEMI [4,5]. Therefore, identifying reliable prognostic indicators within this high-risk subgroup is crucial to enhance risk stratification strategies and optimize therapeutic interventions.

The Systemic Immune Inflammatory Index (SII) is a novel marker derived from peripheral blood cell counts that reflects the inflammatory and immune status of the host by combining neutrophil, lymphocyte, and platelet counts [6,7]. Neutrophils play a crucial role in the initial inflammatory response, while lymphocytes regulate the inflammatory process, and platelets promote thrombosis and are also involved in inflammatory and repair processes. Therefore, SII represents the intricate balance between inflammation, immunity, and thrombosis, all of which are critical in the pathophysiology of ACS. Chronic inflammation concurrently exacerbates disease progression and its complications by inducing insulin resistance, impairing β-cell function, and disrupting glucose metabolism [8].

Previous studies have demonstrated a close association between SII and prognosis in various malignant tumors [9,10]. High SII levels are particularly linked to poor prognosis in solid tumors, hepatocellular carcinoma, lung cancer, and breast cancer [[11], [12], [13]]. Elevated SII has recently been identified as a prognostic indicator linked to adverse outcomes in patients with various cardiovascular diseases (e.g., atrial fibrillation, chronic heart failure, and coronary artery disease), diabetes, and associated complications [[14], [15], [16], [17], [18], [19]]. However, the predictive value of SII in patients with NSTEMI combined with T2DM has not been explored. Considering the intricate interplay among inflammation, immune dysregulation, and metabolic disorders in this cohort, elucidating the association between SII and patient mortality risk holds substantial clinical relevance.

This study aimed to assess the predictive utility of SII in determining the risk of mortality among patients with NSTEMI and T2DM. Through post-stratification analysis based on SII levels, the study sought to ascertain whether SII could serve as a dependable biomarker for risk stratification in this patient population, potentially enhancing therapeutic decision-making processes.

## 2 Methods

### 2.1 Study population and data collection

In this study, data were collected from patients diagnosed with NSTEMI and T2DM between December 2017 and May 2022 in Yichang Central People's Hospital (The First College of Clinical Medical Science, China Three Gorges University). The diagnosis of NSTEMI was established based on clinical presentation with ischemic symptoms and elevated cardiac troponin I (CTnI) levels without ST-segment elevation [20]. The diagnostic criteria for diabetes mellitus included: (1) previously diagnosed diabetes mellitus with glucose-lowering medication (diet, oral medication, and/or insulin); (2) typical symptoms of diabetes mellitus with a random glucose level of ≥11.1 mmol/L, and/or fasting glucose level of ≥7.0 mmol/L, and/or 2-h blood glucose level of ≥11.1 mmol/L after an oral glucose tolerance test; and (3) glycated hemoglobin A1c (HbA1c) level of ≥6.5 % upon admission [21]. Patients who were lost to follow-up, those with missing total lymphocyte counts, and those known to have malignancy, active inflammation, or advanced hepatic or renal failure were excluded from the study. Ultimately, a total of 448 patients were included in the analysis. SII was calculated using the following formula: SII = Platelet count × Neutrophil count/Lymphocyte count. Based on the optimal threshold value of SII determined by the time-dependent receiver operating curve (ROC), the patients were categorized into two groups: the low SII group and the high SII group.

### 2.2 Data collection and study endpoints

Demographic data and relevant clinical history were extracted from the electronic medical record system. Upon admission, blood samples were obtained from each patient. Furthermore, any deaths that occurred during hospitalization or after discharge were meticulously recorded.

### 2.3 Statistical analysis

Statistical analyses were performed using R software (version 4.3.2, R Foundation for Statistical Computing, Vienna, Austria). All statistical tests were two-tailed, with a significance level set at *P* < 0.05. Categorical variables were reported as percentages (number) and analyzed using the chi-square test. Continuous variables were presented as either mean with standard deviation or median with interquartile range (IQR). The normality of continuous variables was assessed using the Kolmogorov-Smirnov test or histograms. For normally distributed data, a two-sample *t*-test was employed, while for non-normally distributed data, the Kruskal-Wallis Rank Sum Test was conducted. The optimal critical value of SII was determined using ROC analysis over time. Univariate and multivariate Cox proportional hazards regression models were used to estimate the hazard ratio (HR) and its 95 % confidence interval (CI). The cumulative risk of the endpoint over time was visually depicted using Kaplan-Meier curves, and the log-rank test was employed to compare the two groups.

## 3 Results

### 3.1 Baseline characteristics

**Fig. 1:**
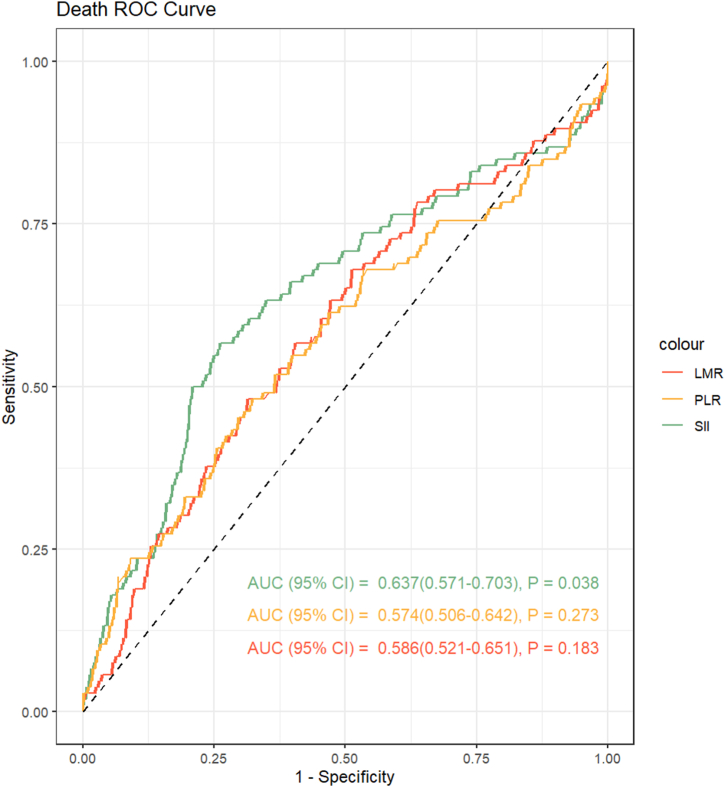
Receiver operating characteristic curve (ROC) analysis with the area under the curve of systemic immune-inflammation index (SII) in predicting mortality.

This study included a total of 448 patient cases (mean age: 65.0 ± 11.2 years, 68.5 % male) with NSTEMI and T2DM. The optimal threshold for SII was determined by analyzing all-cause mortality. As shown in Fig. 1, SII had a good predictive ability for death compared with LMR (lymphocyte-to-monocyte ratio) and PLR (platelet-to-lymphocyte ratio). The ROC analysis evaluated the SII cutoff point for predicting death (1384.596 × 10^9^/L). The study population was divided into two groups based on the SII cutoff value. Table 1 presents the baseline characteristics of patients categorized according to their SII values. The high SII group consisted of older patients who exhibited a higher incidence of acute heart failure, cardiogenic shock in comparison to the low SII group. Patients in the high SII group showed significantly elevated levels of white blood cell counts, platelet counts, neutrophil counts, serum creatinine, LDH, αHBDH, CK, and CK-MB, as well as significantly decreased lymphocyte counts, Hemoglobin, and albumin. Furthermore, the high SII group exhibited a lower percentage of patients receiving treatment with stent, ACEI, biguanides, and sulfonylureas.

**Table 1:** Baseline characteristics of patients classified by SII level.

Variables	Overall (N = 448)	SII level: Low (N = 324)	SII level: High (N = 124)	P value
**Clinical characteristics**				
Age, years (mean [SD])	65.0 (11.2)	63.3 (10.9)	69.3 (10.8)	<0.001
Male sex, n (%)	307 (68.5)	230 (71.0)	77 (62.1)	0.089
Hypertension, n (%)	313 (69.9)	219 (67.6)	94 (75.8)	0.114
Hyperlipidemia, n (%)	64 (14.3)	51 (15.7)	13 (10.5)	0.203
Renal dysfunction, n (%)	49 (10.9)	30 (9.3)	19 (15.3)	0.095
Atrial fibrillation, n (%)	33 (7.4)	21 (6.5)	12 (9.7)	0.339
Hyperuricemia, n (%)	36 (8.0)	29 (9.0)	7 (5.6)	0.338
Stroke, n (%)	53 (11.8)	35 (10.8)	18 (14.5)	0.355
Acute Heart Failure, n (%)	51 (11.4)	21 (6.5)	30 (24.2)	<0.001
Cardiogenic Shock, n (%)	20 (4.5)	10 (3.1)	10 (8.1)	0.043
Cardiac Arrest, n (%)	4 (0.9)	2 (0.6)	2 (1.6)	0.659
Malignant Ventricular Arrhythmia, n (%)	6 (1.3)	2 (0.6)	4 (3.2)	0.091
High AVB, n (%)	9 (2.0)	6 (1.9)	3 (2.4)	0.995
Cardiac Rupture, n (%)	1 (0.2)	0 (0.0)	1 (0.8)	0.617
Ventricular Aneurysm, n (%)	4 (0.9)	2 (0.6)	2 (1.6)	0.659
**Laboratory parameters**				
WBC, 10^9/L (median [IQR])	8.2 [6.4, 10.3]	7.2 [6.1, 9.2]	10.7 [9.0, 13.0]	<0.001
Lymphocyte, 10^9/L (median [IQR])	1.3 [1.0, 1.7]	1.5 [1.2, 1.9]	0.8 [0.6, 1.1]	<0.001
Monocyte, 10^9/L (median [IQR])	0.6 [0.4, 0.8]	0.6 [0.4, 0.7]	0.6 [0.3, 0.8]	0.885
Hemoglobin, g/L (median [IQR])	126.0 [110.0, 139.0]	127.5 [114.8, 141.0]	116.0 [95.0, 136.0]	<0.001
Platelet, 10^9/L (median [IQR])	180.0 [143.0, 223.0]	170.0 [135.0, 209.0]	211.5 [175.2, 258.2]	<0.001
Neutrophil, 10^9/L (median [IQR])	5.9 [4.3, 8.1]	4.9 [3.7, 6.4]	9.2 [7.5, 11.4]	<0.001
ALT, U/L (median [IQR])	24.0 [16.0, 37.2]	24.0 [16.8, 37.0]	22.5 [15.0, 39.2]	0.464
AST, U/L (median [IQR])	36.0 [23.0, 71.0]	33.0 [22.0, 68.0]	43.0 [26.0, 77.0]	0.083
Glucose, mmol/L (median [IQR])	8.0 [5.5, 11.5]	7.4 [5.4, 10.8]	9.2 [6.4, 13.5]	0.001
TG, mmol/L (median [IQR])	1.4 [0.9, 1.9]	1.4 [1.0, 2.0]	1.2 [0.8, 1.7]	0.001
TC, mmol/L (mean [SD])	3.9 (1.5)	3.9 (1.5)	3.8 (1.6)	0.508
LDL-C, mmol/L (mean [SD])	2.3 (1.1)	2.3 (1.1)	2.3 (1.1)	0.896
HDL-C, mmol/L (mean [SD])	1.1 (0.4)	1.1 (0.4)	1.1 (0.5)	0.594
Serum creatinine, μmol/L (median [IQR])	88.0 [71.0, 122.7]	84.0 [69.8, 110.1]	100.0 [77.1, 185.4]	<0.001
Albumin, g/L (median [IQR])	37.0 [34.0, 39.4]	37.4 [34.6, 39.5]	35.8 [32.1, 39.2]	0.001
LDH, IU/L (median [IQR])	275.0 [214.0, 376.2]	254.0 [201.0, 353.8]	321.0 [257.5, 436.5]	<0.001
αHBDH, IU/L (median [IQR])	209.5 [159.0, 305.2]	194.0 [151.0, 289.2]	247.5 [187.2, 368.8]	<0.001
CK, IU/L (median [IQR])	189.5 [87.8, 463.8]	172.0 [80.0, 437.2]	232.0 [128.5, 528.0]	0.008
CK-MB, ng/mL (median [IQR])	21.0 [12.8, 43.0]	19.0 [11.0, 40.0]	28.0 [16.0, 46.5]	<0.001
**Lesions treatment**				
Stent, n (%)	227 (50.7)	198 (61.1)	29 (23.4)	<0.001
Balloon, n (%)	15 (3.3)	12 (3.7)	3 (2.4)	0.702
CABG, n (%)	2 (0.4)	1 (0.3)	1 (0.8)	1.000
SCA, n (%)	37 (8.3)	31 (9.6)	6 (4.8)	0.151
No treatment, n (%)	160 (35.7)	76 (23.5)	84 (67.7)	<0.001
CTA, n (%)	7 (1.6)	6 (1.9)	1 (0.8)	0.710
**Medications**				
Aspirin, n (%)	427 (95.3)	311 (96.0)	116 (93.5)	0.399
Statin, n (%)	444 (99.1)	322 (99.4)	122 (98.4)	0.659
ACEI, n (%)	156 (34.8)	123 (38.0)	33 (26.6)	0.032
ARB, n (%)	135 (30.1)	103 (31.8)	32 (25.8)	0.263
β-blocker, n (%)	349 (77.9)	252 (77.8)	97 (78.2)	1.000
Clopidogrel, n (%)	390 (87.1)	279 (86.1)	111 (89.5)	0.422
Diuretics, n (%)	254 (56.7)	150 (46.3)	104 (83.9)	<0.001
Glycosidase Inhibitors, n (%)	267 (59.6)	202 (62.3)	65 (52.4)	0.071
Biguanides, n (%)	110 (24.6)	95 (29.3)	15 (12.1)	<0.001
DDP-4 Inhibitor, n (%)	104 (23.2)	80 (24.7)	24 (19.4)	0.284
Sulfonylureas, n (%)	185 (41.3)	146 (45.1)	39 (31.5)	0.012
Thiazolidinediones, n (%)	16 (3.6)	14 (4.3)	2 (1.6)	0.272
Subcutaneous Insulin, n (%)	179 (40.0)	114 (35.2)	65 (52.4)	0.001
Sacubitril Valsartan, n (%)	389 (86.8)	273 (84.3)	116 (93.5)	0.014

Numbers are No. (%) unless otherwise noted.SD = standard deviation, fmol/L = femtomole per liter, IQR = interquartile range.

Abbreviations: SII, Systemic immune-inflammation index; AVB, Atrioventricular Block; WBC, White Blood Cell; TG, Triglycerides; TC, Total cholesterol; HDL-C, High-density lipoprotein cholesterol; LDL-C, Low-density lipoprotein cholesterol; LDH, Lactate Dehydrogenase; αHBDH, Alpha-Hydroxybutyrate Dehydrogenase; CK, Creatine Kinase; CABG, Coronary Artery Bypass Grafting; SCA, Percutaneous Coronary Angioplasty; CTA, Computed Tomography Angiography; ACEI, Angiotensin-converting enzyme inhibitor; ARB, Angiotensin receptor blocker.

### 3.2 Comparison of SII-based survival analyses in stratified groups

**Fig. 2:**
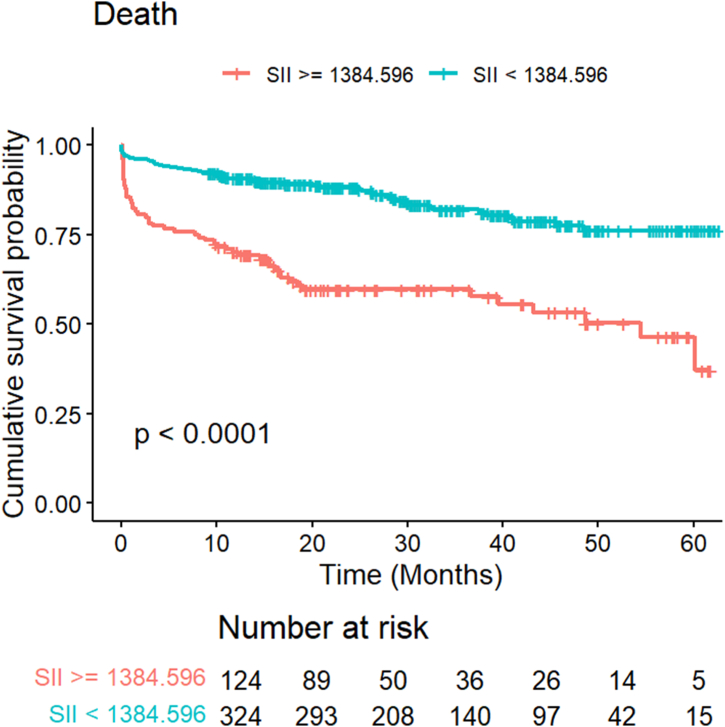
Kaplan-Meier survival curve analysis showing all-caused death.

During a mean follow-up period of 22.75 months, a total of 106 patients died (Table 2). The high SII group exhibited a higher prevalence of mortality compared to the low SII group (42.7 % vs. 16.4 %, *P* < 0.001). The Kaplan-Meier survival curves further demonstrated that patients with elevated SII values had a considerably greater risk of experiencing all-cause mortality (Fig. 2). These findings indicate that a higher SII value is linked to an elevated risk of developing death.

**Table 2:** Mortality in patients according to SII level.

Variables	Overall	SII level: High	SII level: Low	P value
No.	448	124	324	
All cause death, n (%)	106 (23.7)	53 (42.7)	53 (16.4)	<0.001

### 3.3 Multivariate analysis of survival in patients with NSTEMI combined with T2DM

We conducted univariate and multivariate COX regression analyses to determine the independent indicators of all-cause death in patients diagnosed with NSTEMI and T2DM (Table 3). COX regression analyses demonstrated that SII ≥1384.596 × 10^9^/L (HR: 3.190; 95 % CI: (2.180–4.680); *P* < 0.001) was an independent predictor. Furthermore, after conducting our multifactorial analysis and accounting for other pertinent factors, we found that the SII remained significantly correlated with the risk of survival (HR: 2.720; 95 % CI: 1.810–4.090; *P* < 0.001).

**Table 3:** Univariate and multivariate cox regression analysis results for all-cause death.

Variables	Univariate		Multivariate	
	HR (95 % CI)	p-value	HR (95 % CI)	p-value
Sex	0.490 (0.334–0.720)	<0.001	0.680 (0.450–1.010)	0.057
Age	2.990 (1.920–4.650)	<0.001	2.260 (1.430–3.570)	<0.001
Hypertension	1.260 (0.813–1.950)	0.303		
Hyperlipidemia	0.353 (0.155–0.806)	0.013	0.610 (0.270–1.410)	0.249
Aspirin	0.232 (0.123–0.436)	<0.001	0.360 (0.190–0.690)	0.002
Statin	0.141 (0.044–0.446)	0.001	0.250 (0.070–0.870)	0.029
ACEI	0.377 (0.237–0.601)	<0.001	0.490 (0.300–0.780)	0.003
ARB	0.928 (0.597–1.440)	0.742		
β-blocker	0.630 (0.411–0.967)	0.034	0.890 (0.560–1.400)	0.603
SII	3.190 (2.180–4.680)	<0.001	2.720 (1.810–4.090)	<0.001

**Fig. 3:**
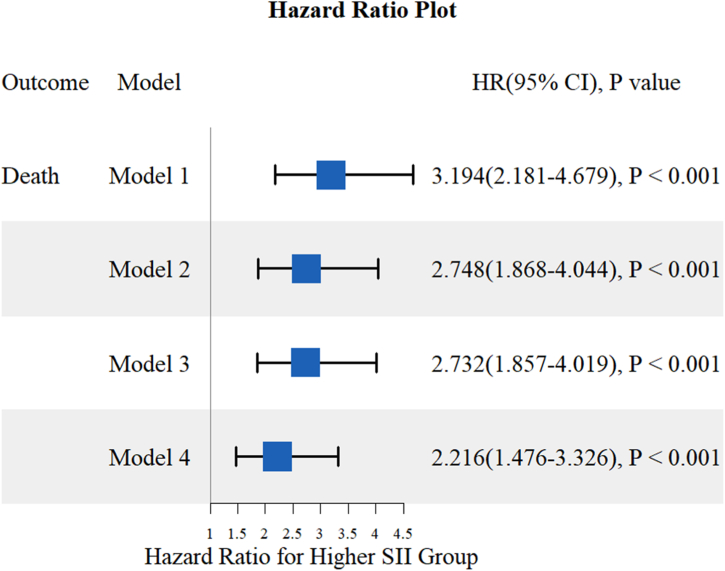
The association of high SII and all-cause death in patients. Notes: Model 1: unadjusted. Model 2: adjusted for gender, age. Model 3: adjusted for gender, age, hypertension, dyslipidemia. Model 4: adjusted for gender, age, hypertension, dyslipidemia, aspirin, Statin, ACEI, ARB, β-blocker.

Fig. 3 displays the results of COX proportional risk regression modeling analyses conducted using four different models to identify independent predictors of mortality. The results showed that high levels of SII were independently associated with nd all-cause death. Furthermore, after adjusting for gender, age, hypertension, dyslipidemia, aspirin use, statin use, ACEI use, ARB use, and β-blocker drug use, high SII levels remained significantly associated with all-cause death (HR: 2.216; 95 % CI: 1.476–3.326; *P* < 0.001).

The C-index, also known as the concordance index, is a widely used measure to evaluate the predictive accuracy of survival analysis models, including Cox proportional risk models. When SII was added to the basic model that included conventional risk factors, there was a slight improvement in the C-index (Table 4). This finding indicates that SII can enhance the predictive value for all-cause death.

**Table 4:** Evaluation of predictive models for all-cause death.

Death	C-Index (95 % CI)
Traditional risk factors	0.739 (0.693–0.786)
Traditional risk factors + SII	0.768 (0.726–0.809)

Notes: Traditional risk factors model: gender, age, hypertension, dyslipidemia, aspirin, Statin, ACEI, ARB, β-blocker.

Abbreviations: C-index, concordance index.

## 4 Discussion

This retrospective study aimed to investigate the predictive value of SII for the risk of death in patients with NSTEMI and T2DM. ROC curve analysis was employed to determine the optimal threshold of SII for predicting mortality. Elevated SII levels were associated with an increased likelihood of death, establishing a potential clinical reference point for identifying patients at heightened risk of adverse outcomes and guiding appropriate interventions. Furthermore, Kaplan-Meier analyses revealed that patients with low SII levels exhibited higher survival rates compared to those with high SII levels, reinforcing the prognostic significance of SII. Univariate and multivariate COX regression analyses demonstrated an independent correlation between elevated SII levels and the risk of death in patients with concurrent NSTEMI and T2DM. Notably, even after adjusting for confounding variables such as age, sex, and comorbidities, elevated SII values remained independently associated with poor prognosis. These results suggest that SII may offer additional prognostic insights beyond conventional risk factors.

Inflammation is increasingly recognized as a pivotal factor in the pathogenesis and prognosis of two prevalent conditions, namely NSTEMI and T2DM. NSTEMI typically involves the rupture of atherosclerotic plaques, constituting a primary pathogenic mechanism underlying myocardial infarction. Inflammation's presence can expedite the formation, progression, and destabilization of plaques, thereby heightening the risk of rupture. Subsequent to plaque rupture, rapid platelet aggregation and thrombosis may obstruct coronary blood flow, culminating in myocardial infarction [22]. At the same time, individuals with T2DM frequently exhibit a chronic low-grade systemic inflammatory state intricately connected with insulin resistance. Insulin resistance induces elevated blood glucose levels, thereby exacerbating the inflammatory response and perpetuating a detrimental cycle. Inflammatory mediators like TNF-α and IL-1β can impede insulin signaling pathways, intensifying insulin resistance and ultimately contributing to the advancement of T2DM and the onset of associated complications [8]. Consequently, modulating the inflammatory response emerges as a critical strategy in the prevention and management of these ailments.

SII is a composite measure that incorporates multiple indicators of inflammation and immune response, providing insight into the overall physiological condition of an individual. Inflammation within the body leads to abnormal platelet elevation. The adherence of abnormally aggregated platelets to endothelial cells induces local ischemia, hypoxia, microthrombosis, leading to the obstruction of blood vessels [23]. This process results in various malignant clinical events, such as ischemic myocardial infarction, stroke, and peripheral vascular disease. A reduction in lymphocyte count signifies excessive apoptosis, impaired immune function, and reduced immune system capacity, which, in turn, contributes to vascular endothelial dysfunction. This condition leads to aberrant platelet aggregation and thrombosis formation following activation. Neutrophils additionally trigger inflammatory responses, cause coronary artery plaque abnormalities, create atherosclerotic plaque rupture, and increase the likelihood of cardiovascular complications due to thrombosis.

According to many researchers, SII is believed to provide a more comprehensive indication of the inflammatory status in patients compared to PLR, NLR, neutrophil count, and lymphocyte count [24,25]. Zhu et al. conducted a retrospective analysis of the clinical features and laboratory data of patients diagnosed with STEMI. Their findings revealed that SII levels were significantly higher in the group of patients who experienced MACE (Major adverse cardiovascular events) compared to the non-MACE group [26]. Esenboğa et al. demonstrated an independent association between SII levels and the occurrence of the no-reflow phenomenon (NRP) in patients who underwent primary percutaneous coronary intervention (PCI) for STEMI [27]. Furthermore, study identified that elevated SII levels may serve as a predictive indicator for short-term mortality among patients with aortic stenosis (AS) who undergo transcatheter aortic valve implantation (TAVI) [28]. In alignment with these findings, our study observed a significantly higher incidence of MACEs in the high SII group. Unlike traditional biomarkers such as the Naples score (nutritional-inflammation focus) or IMRS (multifactorial risk assessment), SII uniquely integrates both inflammatory (neutrophil-lymphocyte axis) and prothrombotic (platelet-driven) pathways, offering a holistic reflection of the pathophysiological mechanisms underlying MACEs [[29], [30], [31]]. This dual-pathway sensitivity positions SII as a cost-effective and accessible tool for risk stratification in acute and chronic cardiovascular settings [7,32], though its lack of nutritional parameters (vs. PNI) highlights opportunities for complementary use with other indices [33].

Numerous studies have demonstrated the significant involvement of the SII in diabetes-related ailments. Diabetic kidney disease (DKD) and peripheral arterial disease (PAD) represent crucial complications of T2DM, significantly elevating morbidity and mortality rates among affected individuals. Recent investigations have revealed a positive correlation between elevated SII levels and an increased predisposition to DKD among individuals with T2DM. Remarkably, this correlation persisted significantly following adjustments for multiple covariates [34]. Another study investigated the relationship between diverse inflammatory biomarkers, including SII, and PAD in T2DM patients. Findings indicated a positive association between elevated SII levels and both the presence of PAD and its clinical severity [18]. Multifactorial logistic regression analysis suggested that SII could function as an independent risk factor for PAD in T2DM patients [18]. A large-scale study revealed that heightened levels of SII correlated with unfavorable clinical prognoses among diabetic patients undergoing PCI [35]. Additionally, Urbanowicz et al. identified SII as a predictive marker for long-term prognosis in diabetic patients undergoing off-pump coronary artery bypass grafting [17]. These findings underscore the potential of SII as a prognostic marker in individuals with diabetic complications and underscore the significance of inflammation in diabetes-related health issues. Incorporating SII into risk stratification frameworks could guide tailored therapeutic approaches, such as intensified anti-inflammatory regimens or optimization of guideline-directed therapies, to mitigate adverse outcomes in this high-risk population.

In this study, high SII levels were found to be positively associated with increased mortality in patients with NSTEM combined with T2DM. However, our study has several limitations that need to be considered. Firstly, this study was a single-center retrospective study without randomization, which may have been affected by selection bias. Secondly, the study was restricted to Chinese subjects; therefore, additional validation is necessary to generalize these findings to other ethnic groups. Thirdly, the calculation of SII was performed solely at admission without monitoring changes in inflammatory biomarkers over the study period. Consequently, the clinical significance of SII in NSTEM combined with T2DM. patients should be further verified through a multicenter study with an expanded sample size.

## 5 Conclusion

Research indicates a robust correlation between elevated SII values and mortality rates among patients diagnosed with NSTEMI concomitant with T2DM. In contrast to conventional risk factors, SII demonstrates superior predictive efficacy in assessing mortality risk. Serving as a straightforward blood biochemical marker, SII holds significant predictive utility in clinical settings, offering valuable insights for risk assessment and treatment strategy formulation.

## CRediT authorship contribution statement

**Cuiyuan Huang:** Writing – review & editing, Writing – original draft, Visualization, Validation, Supervision, Software, Investigation, Formal analysis, Data curation. **Jiajuan Yang:** Writing – original draft, Methodology, Investigation, Data curation. **Wenqiang Li:** Investigation, Formal analysis. **Li Liu:** Investigation, Formal analysis. **Wei Wang:** Investigation, Formal analysis. **Haiyan Hu:** Software, Data curation. **Jing Zhang:** Supervision, Project administration, Funding acquisition, Conceptualization. **Jian Yang:** Supervision, Software, Project administration, Funding acquisition, Conceptualization.

## Ethics statement

The study adhered to the principles outlined in the Declaration of Helsinki and was approved by the Medical Research Ethics Committee of Yichang Central People's Hospital (approval number: 2021-043-01). Informed consent was waived due to the retrospective nature of the study.

## Funding

This work was supported by 10.13039/501100001809National Natural Science Foundation of China (82300969, 82170418, 82271618, 82471616), 10.13039/501100003819Natural Science Foundation of Hubei Province, China (2022CFA015), Central Guiding Local Science and Technology Development Project (2022BGE237); Key Research and Development Program of Hubei Province (2022BCE001, 2023BCB139); 10.13039/100017958Hubei Provincial Health Commission Project (WJ2023M151).

## Declaration of competing interest

The authors declare the following financial interests/personal relationships which may be considered as potential competing interests: Jian Yang, Jing Zhang, Cuiyuan Huang reports financial support was provided by 10.13039/501100001809National Natural Science Foundation of China. If there are other authors, they declare that they have no known competing financial interests or personal relationships that could have appeared to influence the work reported in this paper.

## Data Availability

Data will be made available on request.
